# A Three-Case Series of Thrombotic Deaths in Patients over 50 with Comorbidities Temporally after modRNA COVID-19 Vaccination

**DOI:** 10.3390/pathogens11040435

**Published:** 2022-04-03

**Authors:** Luca Roncati, Antonio Manenti, Lorenzo Corsi

**Affiliations:** 1 Institute of Pathology, Department of Surgery, Medicine, Dentistry and Morphological Sciences with Interest in Transplantation, Oncology and Regenerative Medicine, University of Modena and Reggio Emilia, 41125 Modena, Italy; 2 Unit of Surgery, Department of Surgery, Medicine, Dentistry and Morphological Sciences with Interest in Transplantation, Oncology and Regenerative Medicine, University of Modena and Reggio Emilia, 41125 Modena, Italy; antonio.manenti@unimore.it; 3 Department of Life Sciences, Section of Pharmacology, University of Modena and Reggio Emilia, 41125 Modena, Italy; lorenzo.corsi@unimore.it; 4 National Institute of Biostructures and Biosystems, Inter-University Consortium, 00136 Rome, Italy

**Keywords:** coronavirus disease 2019 (COVID-19), severe acute respiratory syndrome coronavirus 2 (SARS-CoV-2), nucleoside-modified messenger RNA (modRNA), Comirnaty^®^, Pfizer/BioNTech COVID-19 Vaccine, autopsy, histopathology, thrombosis, platelet factor 4 (PF4), immunohistochemistry

## Abstract

Coronavirus disease 2019 (COVID-19) is the most dramatic pandemic of the new millennium; to counteract it, specific vaccines have been launched in record time under emergency use authorization or conditional marketing authorization by virtue of a favorable risk/benefit balance. Among the various technological platforms, there is that exploiting a nucleoside-modified messenger RNA (modRNA), such as Comirnaty^®^, and that which is adenoviral vector-based. In the ongoing pharmacovigilance, the product information of the latter has been updated about the risk of thrombotic thrombocytopenia, venous thromboembolism without thrombocytopenia and immune thrombocytopenia without thrombosis. However, from an in-depth literature review, the same adverse events can rarely occur with modRNA vaccines too. In support of this, we here report a three-case series of thrombotic deaths in patients over 50 with comorbidities temporally after Comirnaty^®^, investigated by means of post-mortem histopathology and immunohistochemistry. In two out of three cases, the cause of death is traced back to pulmonary microthromboses rich in activated platelets, quite similar morphologically to those described in patients who died from severe COVID-19. Even if remote in the face of millions of administered doses, clinicians should be aware of the possible thrombotic risk also after Comirnaty^®^, in order to avoid a misdiagnosis with potentially lethal consequences. Since COVID-19 vaccines are inoculated in subjects to be protected, maximum attention must be paid to their safety, and prophylactic measures to increase it are always welcome. In light of the evidence, the product information of modRNA COVID-19 vaccines should be updated about the thrombotic risk, as happened for adenoviral vector-based vaccines.

## 1. Introduction

Coronavirus disease 2019 (COVID-19) is the most dramatic pandemic of the new millennium; to counteract it, specific vaccines have been launched in record time under emergency use authorization or conditional marketing authorization by virtue of a favorable risk/benefit balance [1]. 

In the ongoing pharmacovigilance, rare deaths of predominantly female, young or middle-aged adults due to thrombotic thrombocytopenia after Vaxzevria^®^ (previously COVID-19 Vaccine AstraZeneca) have been first reported by three independent research groups from Germany and Austria (Andreas Greinacher et al.) [2], Norway (Nina Schultz et al.) [3] and the United Kingdom (Marie Scully et al.) [4]. They have identified, in the production of pathological platelet-activating antibodies against platelet factor 4 (PF4), the life-threatening trigger of these post-vaccinal adverse events, within six weeks after vaccination [2,3,4]. The Society of Thrombosis and Haemostasis Research has hypothesized that post-vaccination autoantibodies can be formed against platelet antigens as a part of the immune stimulation process [5]. These autoantibodies would unleash massive platelet activation via PF4, resulting in vaccine-induced immune thrombotic thrombocytopenia (VITT) [2,5]; cross-reaction between the vaccine and platelets or PF4 has also been advanced as a potential contributing factor in VITT pathogenesis [2]. For this reason, the product package insert has been updated [6], and Vaxzevria^®^, after a suspension period, has been limited to subjects over 60 in many member states of the European Union or even withdrawn from the vaccination program, as happened in Norway and Denmark [7,8]. The same PF4-mediated serious complications have also occurred with another adenoviral vector-based vaccine, which is COVID-19 Vaccine Janssen by Johnson & Johnson [9].

VITT can manifest itself as venous thrombosis, including unusual sites such as the cerebral venous sinus and splanchnic vein, as well as arterial thrombosis [6]. Therefore, the gold-standard criteria that must all be met for a definitive diagnosis of VITT are, to date, the following: (I) COVID-19 vaccine 4 to 42 days prior to symptom onset; (II) any venous or arterial thrombosis (often cerebral or abdominal); (III) thrombocytopenia (platelet count < 150 × 10^3^/mm^3^); (IV) positive PF4 heparin-induced thrombocytopenia (HIT) enzyme-linked immunosorbent assay (ELISA); (V) markedly elevated D-dimer (>4 times upper limit of normal) [10]. Among the laboratory tests today available for the detection of pathological platelet-activating antibodies against PF4 in VITT, HIT ELISA is the one with the greatest reliability and validity [11], and with the most appropriate sensitivity, being around 95% [12]. 

Also known as C-X-C motif chemokine ligand 4 (CXCL4), PF4 is a 70-amino-acid protein released from α-granules of activated platelets during their aggregation [13]. Its major physiologic role appears to be the neutralization of heparin and heparinoids on the endothelial surface of blood vessels, thereby inhibiting local antithrombin activity and promoting coagulation [13]. In this regard, the heparin–PF4 complex is the antigen in the above-mentioned HIT, an idiosyncratic autoimmune reaction to heparin administration [14]. As a strong chemoattractant for neutrophils, monocytes and fibroblasts, PF4 works inside inflammatory processes and wound repair too [13]. 

Besides VITT, venous thromboembolism without thrombocytopenia and immune thrombocytopenia without thrombosis are counted among the possible coagulation disorders of adenoviral vector-based COVID-19 vaccines [6,15]. However, they are not formally listed among the possible adverse events of the other important class of COVID-19 vaccines, namely nucleoside-modified messenger RNA (modRNA) vaccines.

## 2. Case Series

### 2.1. Case #1

**Figure 1:**
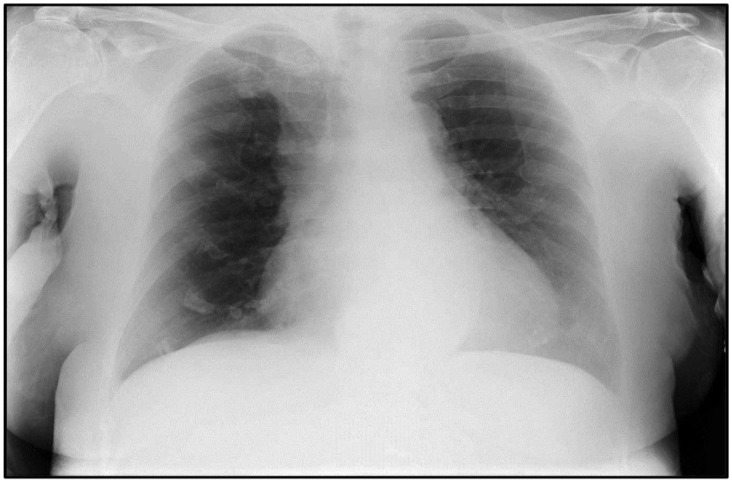
Chest X-ray of case #1 showing left ventricular procidentia with no sign of SARS-CoV-2 pneumonia.

An 81-year-old woman, hypertensive and dyslipidemic in therapy with calcium channel blocker, beta-blocker and statin, allergic to corticosteroids, was admitted to the emergency room for exertional dyspnea with progressive worsening; of note, the patient had received the first dose of Comirnaty^®^ 16 days earlier. Upon admission, the molecular nasopharyngeal swab for the search of severe acute respiratory syndrome coronavirus 2 (SARS-CoV-2) was negative, and the chest X-ray showed only left ventricular procidentia, with no sign of SARS-CoV-2 pneumonia (Figure 1). Blood tests revealed a high D-dimer value (3350 ηg/mL) and severe hypohemoglobinemia (6.9 g/dL) accompanied by low mean corpuscular hemoglobin (16.2 ρg), low mean corpuscular hemoglobin concentration (26.0 g/dL), low mean corpuscular volume (62.1 fL) and low hematocrit (26.4%), while erythrocyte (4.25 × 10^6^/mm^3^), platelet (294 × 10^3^/mm^3^) and white blood cell (7.29 × 10^3^/mm^3^) counts were within normal ranges. For this, on the same day, the patient was submitted to blood transfusion with two units of leukocyte-depleted red cell concentrate. Two days later—that is, 18 days after Comirnaty^®^ administration—the patient was found on the floor in the bathroom of the hospital room in asystole; the subsequent resuscitation maneuvers failed, and an autopsy was required. Blood tests carried out shortly before death highlighted an increase in D-dimer level (5890 ηg/mL) and in post-transfusion hemoglobin (8.8 g/dL), with low sideremia (13 µg/dL), low ferritinemia (4 ηg/mL), qualitatively normal hemoglobin fractions and a slightly decreased platelet count if compared to previous tests (221 × 10^3^/mm^3^).

**Figure 2:**
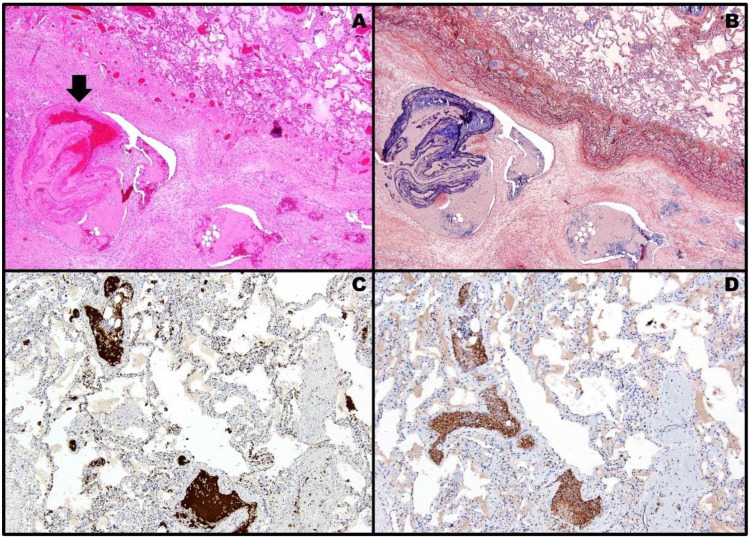
Post-mortem histopathology of case #1 showing thrombotic phenomena inside the lung micro-/macrocirculation: a voluminous thrombus occluding the vascular lumen is noticeable on H&E slide [(**A**), black arrow, 4× objective]. PTAH stains in dark blue the fibrin component of the same thrombus [(**B**), 4× objective]. In the near adjacent lung parenchyma, immunohistochemistry for CD61 [(**C**), 10× objective; antigen retrieval: 64 min] and PF4 [(**D**), 10× objective; antigen retrieval: 36 min] reveals brown-stained plugs of activated platelets within capillaries and small vessels. The semi-quantitative analysis shows a complete (100%) positive overlapping between the staining patterns of the two antibodies.

Surprisingly, post-mortem examination of formalin-fixed paraffin-embedded specimens revealed, by means of hematoxylin and eosin (H&E) and phosphotungstic acid hematoxylin (PTAH) stains, widespread thrombotic phenomena in the micro-/macrocirculation of both the lungs (Figure 2A,B). Therefore, after deparaffinization, hydration, endogenous peroxidase blocking and heat-induced antigen retrieval, the tissue sections were incubated for 32 min at room temperature with anti-CD61 (clone 2f2, prediluted, Ventana), which specifically labels platelets and megakaryocytes, and with the aforementioned anti-PF4 (clone EPR7763, dilution 1:250, Abcam); biotinylated secondary antibody was applied, and the staining product detected with avidin–biotin complex against a hematoxylin counterstain. Detection of the staining reaction was achieved by an enzyme-conjugated polymer complex adapted for an automatic immunostainer from Ventana, with 3,3′-diaminobenzidine tetrahydrochloride as a brownish chromogen; the immunohistochemical results were evaluated by a semi-quantitative method comparing the staining patterns of the two antibodies. Immunohistochemistry confirmed the presence of a large number of activated platelets inside the thrombi (Figure 2C,D).

Unfortunately, it was not possible to search also for anti-PF4 antibodies in the blood of the deceased by HIT ELISA, since more than 10 days had passed from her last blood draw, the maximum storage time of the tubes, until the first autopsy slides were available for preliminary microscopic examination.

### 2.2. Case #2

**Figure 3:**
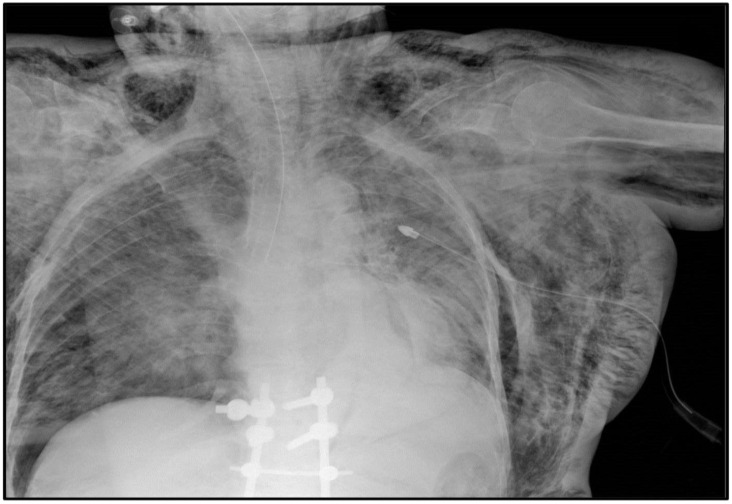
Chest X-ray of case #2 showing bilateral pneumothorax, pneumomediastinum and massive subcutaneous thoraco-abdominal emphysema extended to the upper limbs and neck; a left pleural drainage, the orotracheal tube and spinal surgery outcomes are well observable too.

An 84-year-old woman, affected by autoimmune hemolytic anemia (AIHA) and hypertension in sartan therapy, with a history of vertebral collapses, was admitted to the emergency room for malaise and dyspnea accompanied by desaturation in room air. The patient had received the first and second dose of Comirnaty^®^ 143 and 122 days before, respectively. The chest X-ray taken at the entrance to the hospital showed parenchymal thickening at the suprabasal area of the left paracardiac with ipsilateral pleurogenic haze; the molecular nasopharyngeal swab for SARS-CoV-2 was negative. However, the chest X-ray taken on the next day, when the patient was already in cardiac arrest, pointed out bilateral apico-lateral pneumothorax, pneumomediastinum and massive subcutaneous thoraco-abdominal emphysema extended to the upper limbs and neck (Figure 3). The blood tests performed the same day highlighted severe anemia (hemoglobin: 7.8 g/dL; hematocrit: 21%; erythrocytes: 2.16 × 10^6^/mm^3^), 26.90% of reticulocytes, a high value of lactate dehydrogenase (1869 U/L), mild hyperbilirubinemia (2.84 mg/dL; conjugated: 0.42 mg/dL) and an increase in white blood cell count (15.11 × 10^3^/mm^3^), mainly neutrophils (10.82 × 10^3^/mm^3^). The peripheral blood smear confirmed the presence of numerous spherocytes and disclosed some Howell–Jolly and Pappenheimer bodies; there was no evidence of thrombocytopenia (platelets: 327 × 10^3^/mm^3^).

**Figure 4:**
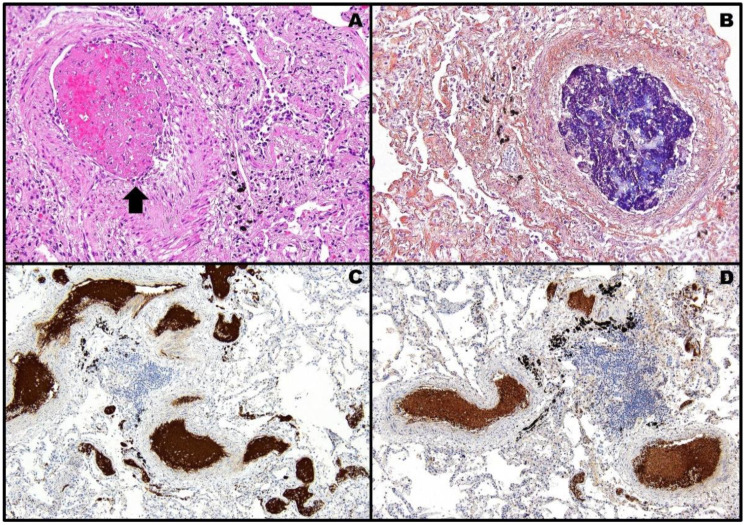
Post-mortem histopathology of case #2 showing thrombotic phenomena inside the lung microcirculation: on H&E slide, an arteriole is fully occluded by a thrombus [(**A**), black arrow, 20× objective]. PTAH stains in dark blue the fibrin core of the same thrombus [(**B**), 20× objective]. Immunohistochemistry for CD61 [(**C**), 10× objective; antigen retrieval: 64 min] and PF4 [(**D**), 10× objective; antigen retrieval: 36 min] highlights widespread brown-stained microthromboses due to activated platelets within the vascular lumens. The semi-quantitative analysis shows a complete (100%) positive overlapping between the staining patterns of the two antibodies.

By means of H&E, PTAH, anti-CD61 and anti-PF4 staining method, performed as for case #1, on post-mortem lung examination, the cause of pneumothorax was traced back to multiple thromboembolic phenomena inside the pulmonary microcirculation even in this circumstance (Figure 4).

### 2.3. Case #3

**Figure 5:**
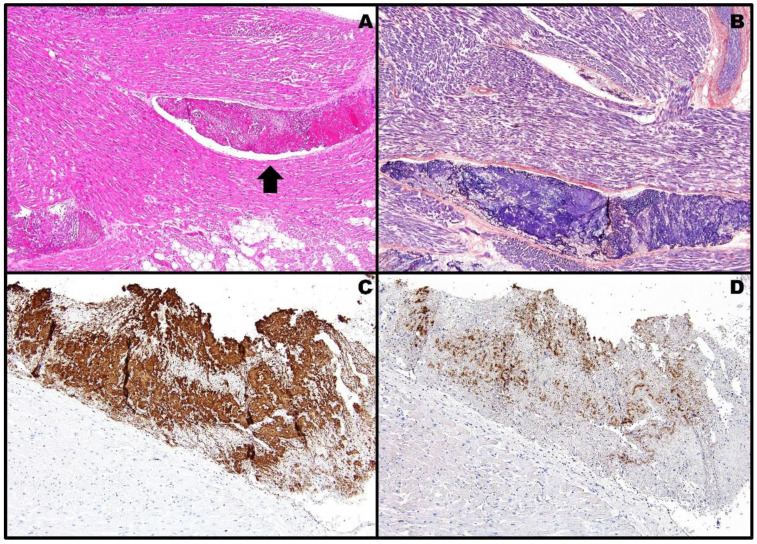
Post-mortem histopathology of case #3 showing sub-endocardial thrombosis: on H&E slide, a thrombus fully fills a sub-endocardial vessel [(**A**), black arrow, 4× objective]. PTAH stains in dark blue the fibrin component of the same thrombus [(**B**), 5× objective]. Immunohistochemistry for CD61 [(**C**), 20× objective; antigen retrieval: 64 min] and PF4 [(**D**), 20× objective; antigen retrieval: 36 min] reveals brown-stained platelets, only partially activated, inside the core of the thrombus. The semi-quantitative analysis in fact shows a partial (30–35%) positive overlapping between the staining patterns of the two antibodies.

A 52-year-old man, thyroidectomized for papillary microcarcinoma and a kidney transplant recipient for polycystic disease undergoing peritoneal dialysis plus tacrolimus in therapeutic range at the last check (7.37 ηg/mL), was found gasping by a relative in his car. Immediately alerted the rescue, the patient died in asystole at the emergency room after 62 min of resuscitation maneuvers. He had received only a single dose of Comirnaty^®^ two weeks and a half before, since four months earlier he had suffered from COVID-19. Before autopsy, a molecular nasopharyngeal swab for SARS-CoV-2 was carried out that was negative; post-mortem histopathology and immunohistochemistry, performed following the same methodological procedure as for case #1 and case #2, revealed mural thrombosis of the right heart ventricle and of a subendocardial vessel with images of organization (Figure 5).

## 3. Discussion

During the COVID-19 pandemic, many technology platforms have been exploited to develop specific and effective vaccines, such as RNA-based, DNA-based, non-replicating viral vector, inactivated virus, live attenuated virus, protein subunit and virus-like particle [16]. Among the next-generation strategies, modRNA vaccines appear the most attractive, since they show superior design, production speed, lower cost of production and no infective risk [16]. 

Comirnaty^®^ by Pfizer/BioNTech/Fosun is a single-stranded 5′-capped modRNA produced using a cell-free in vitro transcription from the corresponding DNA templates of the SARS-CoV-2 spike protein, and then embedded in lipid nanoparticles [17]. However, as with any drug, it can present side effects more or less common and of variable severity, such as: headache (≥1/10), diarrhea (≥1/10), arthralgia and myalgia (≥1/10), injection site pain or swelling (≥1/10), fatigue (≥1/10), chills (≥1/10), pyrexia (≥1/10), nausea and vomiting (≥1/100 to <1/10), injection site redness (≥1/100 to <1/10), lymphadenopathy (≥1/1000 to <1/100), hypersensitivity reactions including rash, pruritus, urticaria or angioedema (≥1/1000 to <1/100), decreased appetite (≥1/1000 to <1/100), insomnia (≥1/1000 to <1/100), lethargy (≥1/1000 to <1/100), hyperhidrosis and night sweats (≥1/1000 to <1/100), pain in extremities (≥1/1000 to <1/100), asthenia and malaise (≥1/1000 to <1/100), injection site pruritus (≥1/1000 to <1/100), acute peripheral facial paralysis (≥1/10,000 to <1/1000), myocarditis and pericarditis (<1/10,000), anaphylaxis (frequency not known), paresthesia and hypoesthesia (frequency not known), erythema multiforme (frequency not known), facial swelling and swelling of the vaccinated limb (frequency not known) [17]. 

Rare thrombotic events have been also reported in relation to Comirnaty^®^ administration, particularly cerebral venous thrombosis [18,19,20,21,22,23,24,25], deep vein thrombosis [26,27,28,29,30,31], retinal vein occlusion [32,33] and coronary thrombosis [34]; moreover, thrombocytopenia and hemorrhage following Comirnaty^®^ have been described [35,36,37,38,39,40,41,42,43,44]. The same coagulation disorders can also occur after Spikevax^®^ (previously COVID-19 Vaccine Moderna), the secondly approved modRNA COVID-19 vaccine on the market [45,46,47,48,49,50]. From a recent analysis of VigiBase, the World Health Organization’s global individual case safety report database and the single largest drug safety data repository today available, a low risk of arterial thrombosis has been found in all vaccines taken into consideration, namely Vaxzevria^®^, Comirnaty^®^ and Spikevax^®^ [51].

Overall VITT incidence following Vaxzevria^®^ has been estimated at around 1 in 80,000 doses or between 10 and 15 cases per million doses [52]; the highest incidence was reported from Norway, in which five cases were described among approximately 130,000 individuals vaccinated by Vaxzevria^®^, suggesting a nationwide incidence of 1 in 26,000 [3]. However, positive HIT ELISA can occur after SARS-CoV-2 vaccination with both adenoviral vector-based and modRNA vaccines [53]: the VITT reporting rate from the United States after modRNA vaccines was 0.00855 per million doses [54]. The median age of VITT patients following adenoviral vector-based vaccination was 44.5 years, with a female predominance, while that after modRNA vaccination was over 50 in both sexes [54]. PF4-dependent platelet-activating antibodies are transient in most patients with VITT [55]; in addition, HIT ELISA reactivity alone has been directly associated with COVID-19 severity in a cohort of 65 hospitalized patients without any evidence of HIT/VITT [56]. 

Here, for the first time in the worldwide literature, we have applied anti-PF4 immunohistochemistry on post-mortem specimens collected from three patients over 50 who temporally died after Comirnaty^®^. Even if anti-PF4 immunohistochemistry cannot be used in VITT assessment since it is highly sensible but unspecific, the detection of CD61-positive PF4-positive platelets clots not only attached to the endothelial surface, where PF4 exerts its physiological action, but also inside the lumen of vessels appears useful to testify to pathological intravascular platelet activation and aggregation. Notably, in the two female patients of our series, the lung histopathological picture was overlapping and characterized by distinctive PF4-rich thrombotic phenomena inside the microcirculation (microthromboses), quite similar from a morphological point of view to those described in patients who have died from severe COVID-19, in which capillary dysfunction has been proven to interfere negatively with blood and tissue oxygenation [57,58,59]. It is reasonable to assume that post-infection or post-vaccination antibodies may behave as platelet-activating factors in rare circumstances, thus generating a hypercoagulable state and an abnormal immunothrombosis, as hypothesized by the Society of Thrombosis and Haemostasis Research [5]; a genetic predisposition to develop such dysimmune/autoimmune complications may be only supposed. It also remains to be clarified how long this state of hypercoagulability can last: if the same morphological picture has been found 18 days after the first dose in case #1 and 143 days after in case #2, it should persist at least up to 4–5 months from the first injection. 

Preexisting conditions could play a synergistic role; for instance, the patient of case #2 was affected by AIHA, nowadays a recognized possible cause of pulmonary embolism [60,61], while the patient of case #3 was a kidney transplant carrier in therapy with tacrolimus, a well-known immunosuppressant seldom responsible for thrombotic microangiopathy [62,63]. The same chronic renal disease generates a biohumoral background at risk for venous thromboembolism [64,65]. Underlying vascular diseases (e.g., atherosclerosis) may impact negatively, too; moreover, the presence of widespread lung microthromboses, particularly those more peripheral, can favor the onset of bilateral pneumothorax [66,67], a further aggravating illness observed in case #2 of our series. 

More than 558 million doses of COVID-19 vaccines were administered in the United States from 14 December 2020 through 21 March 2022 [68]. During this time, the Vaccine Adverse Event Reporting System (VAERS), co-managed by the Centers for Disease Control and Prevention (CDC) and the Food and Drug Administration (FDA), received 13,434 preliminary reports of death among citizens who received a COVID-19 vaccine (0.0024%) [68]. As of 30 January 2022, approximately 570 million doses of Comirnaty^®^ had been administered to people in the European Union [69]: the European database of suspected adverse drug reaction reports (EudraVigilance) contains to date 7023 side effects with a fatal outcome (0.0012%) [69]. The COVID-19 vaccine safety annual report (27 December 2020–26 December 2021) by the Italian Medicines Agency (AIFA) has estimated an overall rate of death reports per 100,000 doses administered equal to 0.70 [70]; this rate falls to 0.66 per Comirnaty^®^ [70].

## 4. Conclusions

Even if remote, clinicians should be aware of the possible thrombotic risk also after Comirnaty^®^ to carefully investigate their patients in the event of clinical suspicion, to promptly initiate antiaggregants or non-heparin anticoagulants as deemed necessary and to avoid a misdiagnosis with potentially lethal consequences. In patients with known capillary dysfunction and not prone to bleeding, we suggest a prophylactic therapy with acetylsalicylic acid at a dosage of 100 mg per day on a full stomach during the peri-vaccination period (at the discretion of the treating physician). Finally, the product information of modRNA COVID-19 vaccines should be updated about this rare risk, as happened for adenoviral vector-based vaccines.

## Data Availability

The data presented in this study are available on reasonable request from the corresponding author. The data are not publicly available due to privacy restrictions.
